# Effects of Cathepsins on Gel Strength and Water-Holding Capacity of Myofibrillar Protein Gels from Bighead Carp (*Aristichthys nobilis*) under a Hydroxyl Radical-Generation Oxidizing System

**DOI:** 10.3390/foods11030330

**Published:** 2022-01-25

**Authors:** Han Lu, Yunhong Liang, Xiangmei Zhang, Gang Wen

**Affiliations:** College of Bioscience and Engineering, Hebei University of Economics and Business, Shijiazhuang 050061, China; liangyunhong1104@163.com (Y.L.); zxm_bio@126.com (X.Z.); harbour_wen@163.com (G.W.)

**Keywords:** bighead carp, cathepsins, gel strength, WHC, hydroxyl radicals

## Abstract

This study investigates the effects of cathepsins on the gel strength and water-holding capacity (WHC) of myofibrillar protein gels from bighead carp (*Aristichthys nobilis*) under a hydroxyl radical-generation oxidizing system. The myofibrillar proteins were divided into control group (with cathepsins) and E64 group (without cathepsins). The changes of cathepsin B and cathepsin L activities, sodium dodecyl sulfate-polyacrylamide gel electrophoresis (SDS-PAGE), protein oxidation (total sulfhydryl and carbonyl contents), and chemical interactions (nonspecific association, ionic bonds, hydrogen bonds, hydrophobic interactions, and disulfides) of myofibrillar protein and gels, as well as the gel strength and WHC of two groups under 0–100 mM H_2_O_2_, were measured. The results indicated that mild oxidation (10 mM H_2_O_2_) made a better gel strength and WHC. Cathepsin B and L activities decreased with increasing H_2_O_2_ concentrations but their effects on myofibrillar protein degradation still existed during 0.1–50 mM H_2_O_2_, which was expressed by higher carbonyl contents and ionic bonds at 0.1 and 50 mM H_2_O_2_, higher total sulfhydryl contents at 0 mM H_2_O_2_, and a lower intensity of MHC and actin of the control group than the E64 group. Besides more protein degradation, cathepsin proteolysis also resulted in lower gel strength and WHC in control gels than E64 gels under mild oxidation, which could be explained by lower hydrophobic interaction and moderate disulfides bonds between gel protein molecules of control gels.

## 1. Introduction

Surimi modori is a problem that cannot be ignored in surimi products, which could result in quality deterioration, and eventually have a negative effect on the acceptance and commercial price of the surimi products [1]. Cathepsins, which belong to a large family of lysosomal cysteine proteases, play a major role in protein degradation of postmortem fish muscle [2]. They hydrolyze a broad range of proteins, such as myosin, actin, nebulin, insulin, myoglobin, glucagon, azocasein, histones, haemoglobin, and insoluble collagen [3,4,5]. The degradation of myofibrillar proteins by endogenous cathepsins is the main cause for surimi modori. There are three reasons for the important role played by endogenous cathepsins on surimi modori: firstly, the cathepsins are myofibril-bound protease and difficult to remove by rinsing during the surimi preparation [6]; secondly, the optical pH value for cathepsin B and L hydrolysis is within the range of postmortem fish (pH = 5–7); thirdly, the cathepsins are heat-stable and exhibit considerable proteolysis rates, especially at 50–70 °C [7], which indicate their possible role in heat-induced surimi gels. Liu, Yin, Zhang, Li and Ma [6] found that the gel strength, breaking force, and deformation were significantly decreased in the groups treated by cathepsin B and L, and cathepsin L likely played a more important role in gel softening. Hu et al. [8] also proved that cathepsin L is involved in modori phenomenon by its hydrolysis of the main protein in carp surimi and results in the decreased gel strength.

In addition, oxidation caused by radicals often occurred in surimi products. Metabolic and other processes occurring in muscle tissue give rise to oxidative compounds, such as hydroxyl radicals, peroxyl radicals, superoxide anions, hydrogen peroxide, and nitric oxide [9,10], which promote the degree of protein oxidation in surimi products. However, there was no agreement about the effects of oxidation on the surimi gel strength and water-holding capacity (WHC). For example, Li et al. [11] reported that the hydroxyl radicals destroyed the springiness, hardness, and WHC of myofibrillar protein gels from common carp. Meanwhile, Lu et al. [12] and Zhou et al. [13] indicated that moderate oxidation could improve the texture as well as WHC of protein gel.

Both cathepsins and myofibrillar proteins in surimi gels have been proved that they can be affected by radical oxidation during postmortem. The gel strength and WHC would be influenced due to the oxidized cathepsins and myofibrillar protein in gels. Cathepsin B and L, which belong to cysteine proteases, were sensitive to oxidation caused by radicals. Their activities would be reduced because cysteine can be oxidized to form sulfenic acid, sulfinic acid, sulfonic acid, or disulfides according to oxidation degree [14]. Therefore the hydrolysis of myofibrillar protein by cathepsins would be weakened and theoretically the gel properties would be promoted. The oxidation-induced changes of myofibrillar protein include loss of sulfhydryl groups, the formation of protein carbonyls, and the formation of crosslinks [11,12]. These changes would influence the ionic bonds, hydrogen bonds, hydrophobic interaction, and disulfides of myofibrillar protein gels, which are also regarded as the molecule forces to maintain the gel network [15]. Lin et al. [16] reported that non-specific association and hydrophobic interactions participate in the generation of the reticular structure in gels, and that disulfide bonds play a decisive role in heat-induced gels of hairtail surimi. Kobayashi et al. [17] also found hydrophobic interactions and disulfide bonds significantly increased during the preparation of tilapia protein and the corresponding surimi gel. The functional properties of myofibrillar protein gels were also related with chemical forces, such as textural properties [18] and WHC [19]. Nevertheless, the effects of oxidation on gel strength and WHC through the chemical interactions changes of myofibrillar protein gels were not obtained.

In addition, the hydrolysis degree of myofibrillar protein by cathepsins also changed due to protein structure under oxidation. According to Bao and Ertbjerg [20], protein structures under moderate oxidation may unfold and spread, which was helpful to proteolysis and thus leads to tender meat, while protein structures under excessive oxidation were more compact; this reduces proteolysis and thereby results in tough meat. Carlin et al. [21] reported that the oxidative changes in the structure of myosin and actin reduce their sensitivity to proteolytic enzymes. Sante-Lhoutellier et al. [22] also reported that oxidation by 1–5 mM H_2_O_2_ in the Fenton reaction may negatively affect the susceptibility of myofibrillar proteins to proteolysis. As a result, there was no agreement on the effects of oxidation on gel strength and WHC when considering cathepsin activity, myofibrillar protein, and cathepsin hydrolysis of myofibrillar protein.

We have two aims in this study: (1) to determine the changes of cathepsin activity and myofibrillar protein under different oxidation condition caused by hydroxyl radicals and whether the oxidized cathepsins still hydrolyze oxidized myofibrillar protein; (2) to determine whether the gel strength and WHC changed accordingly because of the above results and the potential mechanism. Therefore, the hydroxyl radical-generation oxidizing system was used to create an oxidation environment for myofibrillar protein isolated from bighead carp (*Aristichthys nobilis*) fillets with/without cathepsins (control and E64 group, respectively). We measured the changes of cathepsin B and cathepsin L activities, carbonyl content, total sulfhydryl content, sodium dodecyl sulfate-polyacrylamide gel electrophoresis (SDS-PAGE) and chemical interaction (nonspecific association, ionic bonds, hydrogen bonds, hydrophobic interactions, and disulfides) of myofibrillar protein and gels, as well as the gel strength and WHC under different H_2_O_2_ concentrations.

## 2. Materials and Methods

### 2.1. Materials

Thirty-three bighead carps (weight 1438 ± 141 g, length 55 ± 1 cm) were purchased from Beiguo supermarket in Shijiazhuang, Hebei province, China, and transported to the laboratory alive in a box with water and an oxygenation device. The bighead carps were killed immediately by a blow to the head and eviscerated, and the internal organs were removed; then, the blood in the abdominal cavity was washed by flowing tap water. The heads were cut off and one fish body was cut into two fillets. The process was finished in three hours in according with the Laboratory animal-Guideline for ethical review of animal welfare (GB/T 35892-2018) developed by China General Administration of Quality Supervision, Inspection and Quarantine and National Standardization Administration and the dorsal muscle of fillets were used for further analysis.

Six fillets were used for measuring cathepsin activity, and the remaining fillets were used for the extraction of myofibrillar protein used in the hydroxyl radical-generation oxidizing system.

The substrates for cathepsin B (Z-Arg-Arg-MCA, C5429), the substrates for cathepsin B+L (Z-Phe-Arg-MCA, C9521), and the general cysteine proteinase inhibitor E64 (E3132), were all purchased from the Sigma Company, Shanghai, China.

### 2.2. Measurement of Cathepsin B and Cathepsin L Activities in Myofibrils under Hydroxyl Radical-Generation Oxidizing System

Muscle samples (0.5 g) were homogenized by a homogenizer (FM200, Fluko Co., Shanghai, China) with 4.5 mL of buffer (100 mM sucrose, 100 mM KCl, 50 mM tris-HCl, 10 mM sodium pyrophosphate, 1 mM Na_2_EDTA, and pH 7.2) at 15000 rpm for 10 s. The mixture was incubated at 4 °C for 36 h with a hydroxyl radical-generation oxidizing system (10 μM FeCl_3_, 0.1 mM ascorbic acid, 0, 0.1, 0.5, 1, 5, 10, 50, and 100 mM H_2_O_2_, respectively). Myofibrils were the precipitates obtained through centrifuging the homogenate at 1100× *g* for 20 min. Cathepsin B and L activity in myofibrils were measured according to Barrett and Kirschke [23]. Enzyme activation was performed by incubating 500 μL of myofibril fractions (diluted with 0.1% Brij 35 (1/500, *v*/*v*)) with 1250 μL of assay buffer (cathepsin B (pH 6.0): 352 mM KH_2_PO_4_, 48 mM Na_2_HPO_4_, 4 mM Na_2_EDTA, and 8 mM cysteine; cathepsin B + L (pH 6.8): 200 mM KH_2_PO_4_, 200 mM Na_2_HPO_4_, 4 mM Na_2_EDTA, and 8 mM cysteine) for 5 min at 40 °C, and then the reaction was initiated by adding 250 μL of 20 μM substrates (cathepsin B:Z-Arg-Arg-MCA; cathepsin B+L: Z-Phe-Arg-MCA) for 30 min at 40 °C. The reaction was stopped by adding 2 mL of a termination buffer (100 mM sodium chloroacetate, 70 mM acetic acid, and 30 mM NaAc, pH 4.3). Then, samples were measured in triplicate using a fluorescent spectrophotometer (RF-5301PC, SHIMADZU, Japan), and the excitation (λex) and emission (λem) wavelengths were set to 380 and 460 nm, respectively. One unit of enzyme activity is defined as the amount that hydrolyzes 1 mmol of substrate/min at 40 °C.

### 2.3. Preparation of Myofibrillar Protein from Bighead Carp Fillets

The myofibrillar protein was extracted according to Xiong, et al. [24] through homogenizing 5 g muscle and 4 volumes of 10 mM potassium phosphate buffer solution (10 mM KH_2_PO_4_, 10 mM K_2_HPO_4_, pH 6.8, 100 mM NaCl, 2 mM MgCl_2_, and 1 mM EGTA). The mixture was centrifuged and the pellets were washed twice with the buffer solution and once with 100 mM NaCl. The final pellets, regarded as myofibrillar protein, were separated into the control group and E64 group. The myofibrillar protein used in E64 group was prepared as that in the control group but all extracting and washing solutions used were added with 50 µM E64, which would totally inhibit the cathepsin activity of the myofibrillar protein. The protein concentration was determined using the biuret method [25].

### 2.4. Incubation of Myofibrillar Protein in Hydroxyl Radical-Generation Oxidizing System

Myofibrillar protein in the control and E64 groups were suspended in 15 mM piperazine-*N*, *N* bis (2-ethane sulfonic acid) (PIPES) buffer (pH 6.8, 0.6 M NaCl) without and with 50 µM E64, respectively, for 12 h at 4 °C. Then, 30 mg/mL myofibrillar protein was incubated at 4 °C for 36 h with a hydroxyl radical-generation oxidizing system (10 μM FeCl_3_, 0.1 mM ascorbic acid, 0, 0.1, 0.5, 1, 5, 10, 50, and 100 mM H_2_O_2_, respectively) according to Xiong, Blanchard, Ooizumi, and Ma [24]. Oxidation was terminated by washing the mixture with 3 volumes of 15 mM PIPES buffer twice and with cold, deionized water once to remove hydroxyl radicals. The washing PIPES buffer and water used in E64 group were added with 50 µM E64. Therefore, the samples in control group were affected by both hydroxyl radicals and cathepsins, while the samples in the E64 group were only affected by hydroxyl radicals, as E64 inhibited the cathepsin activity. The pellets obtained by centrifugation at 10,000× *g* for 15 min were collected for further analysis in 24 h.

### 2.5. SDS-PAGE

The method of SDS-PAGE was according to Wang et al. [26], with some modifications. A discontinuous SDS-PAGE with 4% stacking gel and 10% running gel was used to determine the myofibrillar protein patterns. The protein (2 mg/mL) mixed with the sample buffer with and without 1 M dithiothreitol (DTT) was boiled for 4 min.

### 2.6. Determination of Protein Oxidation

#### 2.6.1. Carbonyl Content

The carbonyl content of the myofibrillar protein was expressed as an nmol of 2 4-Dinitrophenylhydrazone (DNPH)-fixed myofibrillar protein using an absorption coefficient of 22,000 mol·L^−1^·cm^−1^ for protein hydrazones, according to Oliver et al. [27].

#### 2.6.2. Total Sulfhydryl Contents

The total sulfhydryl contents of myofibrillar protein were measured according to the method of Benjakul et al. [28]. Half a milliliter (0.5 mL) of the myofibrillar protein solution (4 mg/mL) was mixed with 4.5 mL of 0.2 M Tris-HCl buffer (pH 8.0, containing 8 M urea, 1% SDS, and 3 mM EDTA). Four milliliters (4 mL) of the mixture was added to 0.5 mL of 10 mM DTNB and 0.2 M Tris-HCL buffer (pH 8.0), then incubated in the dark at 40 °C for 25 min. The absorbance at 412 nm was determined. A blank was conducted by replacing the sample with 0.6 M NaCl (pH 7.0).

### 2.7. Preparation of Gels and Gel Properties

#### 2.7.1. Preparation of Heat-Induced Gel

The oxidized myofibrillar protein collected was adjusted to 65 mg/mL using 15 mM PIPES buffer (pH 6.8, 0.6 M NaCl) and put in a 50 mL capped centrifuge tube (15.0 cm in length and 3.0 cm in diameter). Then, the gel was conducted through heating the myofibrillar protein at 40 °C for 30 min and then 90 °C for 20 min in a temperature-controlled water bath. After being heated, the gel was placed into ice water immediately for 1 h to cool down prior to analysis.

#### 2.7.2. Gel Strength

The breaking force (g) and deformation distance (mm) of the cut gel cylinders (2 cm high) were measured using a CT-3 texture analyzer (Brookfield, Wis., Middleboro, MA, USA) with a TA-50 probe at a constant speed of 1.0 mm/s and 30% axial compression according to Lu et al. [29]. The gel strength (g·mm) was calculated by multiplying the breaking force and deformation distance.

#### 2.7.3. WHC

The WHC was determined by the centrifugal loss of gels using a centrifugal force of 4500× *g* for 5 min on 3 g of gel, in accordance with Hultmann and Rustad [30]. The centrifugal loss was expressed as the percentage of remained gel weight relative to the original weight.

### 2.8. Chemical Interactions between Myofibrillar Protein/Gels

The chemical interactions between myofibrillar protein/gels were determined using the method of M.C. et al. [31], with some modifications. Myofibrillar protein/gels (3 g) were homogenized with 15 mL 0.05 M NaCl (S1), 0.6 M NaCl (S2), 0.6 M NaCl, 1.5 M urea (S3), 0.6 M NaCl, 8 M urea (S4) and 0.6 M NaCl, 8 M urea, and 0.5 M DTT (S5) and stored at 4 °C for 1 h, respectively. The mixture was centrifuged at 10,000× *g* for 25 min and the protein concentration in supernatant of S1, S2, S3, S4, and S5 was measured by Lowry’s method and regarded as nonspecific association, ionic bonds, hydrogen bonds, hydrophobic interactions, and disulfide bonds between myofibrillar protein/gels, respectively.

### 2.9. Statistical Analysis

All measurements were carried out in triplicate and data were presented as the mean ± standard deviation. Significant difference (*p* < 0.05) between samples in the control or E64 group under different H_2_O_2_ concentrations was defined by the Duncan method in one-way ANOVA. Significant difference (*p* < 0.05) between samples under the control and E64 groups was defined by the LSD method in one-way ANOVA. The statistical results were obtained through calculating by SPSS 20.0 software (version 20.0; SPSS Inc., Chicago, IL, USA)

## 3. Results

### 3.1. Cathepsin B and Cathepsin L Activity

**Figure 1:**
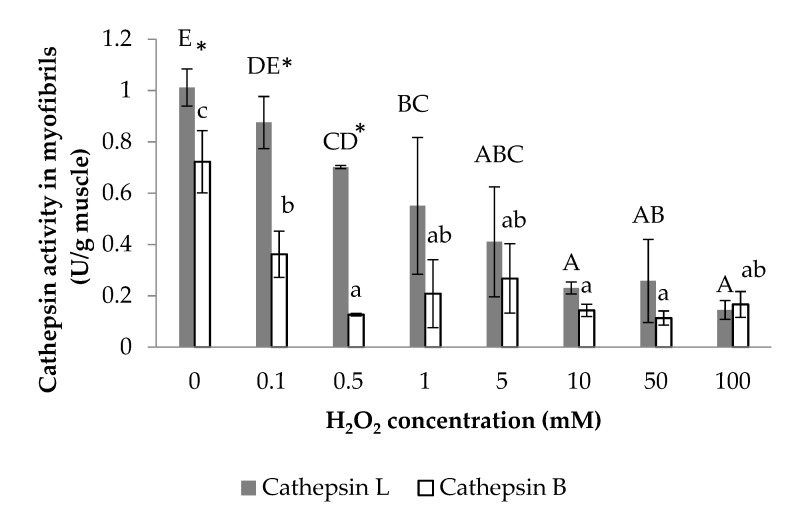
Cathepsin L and cathepsin B activities from bighead carp myofibrils under different H_2_O_2_ concentrations. The same lowercase letter indicates no significant differences between cathepsin B activities under different H_2_O_2_ concentrations. The same capital letter indicates no significant differences between cathepsin L activities under different H_2_O_2_ concentrations. The asterisk indicates significant difference between cathepsin L and cathepsin B activities under the same H_2_O_2_ concentration (*p* < 0.05).

As shown in Figure 1, the cathepsin L activity in myofibrils decreased significantly from 1.01 to 0.14 U/g muscle, as the H_2_O_2_ concentration increased from 0 to 100 mM. Similarly, the cathepsin B activity also decreased significantly when H_2_O_2_ increased from 0.1 to 0.5 mM (*p* < 0.05) and then kept at about 0.15 U/g muscle with no significant difference at 0.5–100 mM H_2_O_2_. Cysteine, as the active site residues of cathepsin B and cathepsin L, was most susceptible to oxidation by all forms of radicals due to their sulfur atoms [32], which could be oxidized by hydroxyl radicals generated by H_2_O_2_ and thus resulted in the decrease of cathepsin activities. In addition, the cathepsin L activity was higher than cathepsin B activity during 0–0.5 mM H_2_O_2_, but there was no significant difference between them during 1–100 mM H_2_O_2_. This result indicated that cathepsin L had greater effects on protein degradation than cathepsin B, which was also reported by Lu et al. [33] in chilled and partial frozen bighead carp.

### 3.2. SDS-PAGE

**Figure 2:**
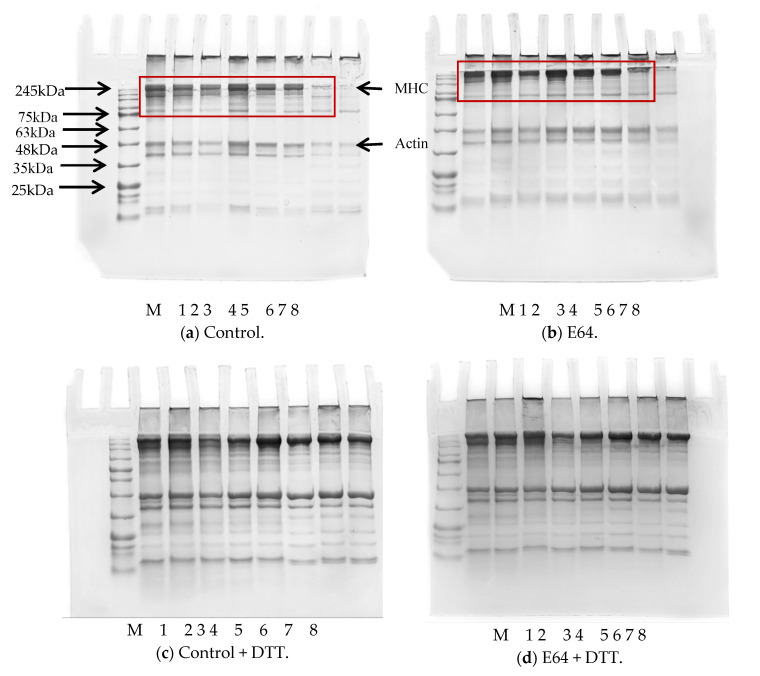
SDS-PAGE profile of myofibrillar protein from bighead carps under different H_2_O_2_ concentrations: (**a**) control (**b**) E64; (**c**) control + DTT; (**d**) E64 + DTT. Lane M: marker; lanes 1–8: samples under 0, 0.1, 0.5, 1, 5, 10, 50, and 100 mM H_2_O_2_, respectively. MHC—myosin heavy chain.

The effects of different H_2_O_2_ concentrations on myofibrillar protein profiles by the SDS-PAGE under reduced and non-reduced conditions were shown in Figure 2. The myosin heavy chain (MHC) and actin intensity of control and E64 groups in Figure 2a,b at 50 and 100 mM H_2_O_2_ were obviously lower than that at 0–10 mM H_2_O_2_. Li et al. [34] also found that MHC and actin decreased gradually with the increase of H_2_O_2_ concentration, especially under high H_2_O_2_ concentration. In addition, there were more protein bands between 75–220 kDa in the control group than that in E64 group at 0–50 mM H_2_O_2_ shown in the red box of Figure 2a,b, which were regarded as the hydrolysis products of MHC by cathepsins existing in control group. A number of in vitro studies have clearly demonstrated the susceptibility of MHC and actin to proteolysis by cathepsins B and L [35,36,37]. Hu et al. [8] reported that cathespin L could hydrolyse MHC and cause rapid decrease of gel strength and flexibility of the heat-induced gel. The effects of cathepsin hydrolysis were also proved by the higher MHC and actin intensity in the E64 group than that in the control group at 0–100 mM H_2_O_2_.

The reduced SDS-PAGE in Figure 2c,d demonstrated much higher intensity of the MHC and actin bands at 50 and 100 mM H_2_O_2_ than that of the non-reduced condition in all groups, which proved the formation of disulfide crosslinking of MHC and actin at 50 and 100 mM H_2_O_2_. In addition, the reduced MHC band intensity at 50 and 100 mM H_2_O_2_ in both groups exhibited dramatic decline compared with the MHC band at 0 and 0.1 mM H_2_O_2_, suggesting that the protein degradation formed during extensive oxidation. There was no significant difference between the control and E64 groups in reduced SDS-PAGE.

### 3.3. Protein Oxidation Parameters

#### 3.3.1. Carbonyl Content

**Figure 3:**
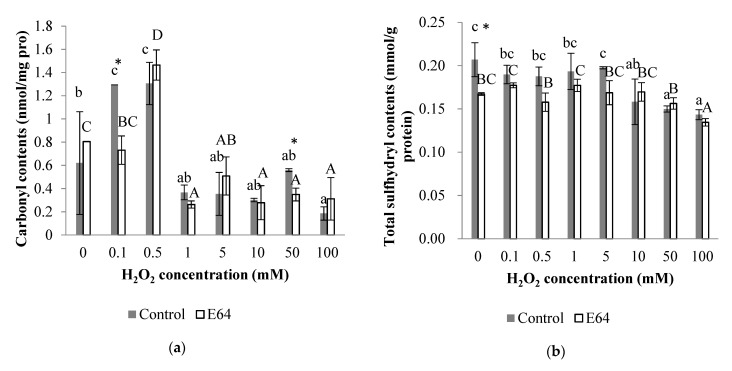
Changes of carbonyl contents (**a**) and total sulfhydryl contents (**b**) of myofibrillar protein from bighead carps under different H_2_O_2_ concentrations. The same lowercase letter indicates no significant differences between samples of control group under different H_2_O_2_ concentrations. The same capital letter indicates no significant differences between samples of E64 group under different H_2_O_2_ concentrations. The asterisk indicates significant difference between samples in control and E64 groups under the same H_2_O_2_ concentration (*p* < 0.05).

As shown in Figure 3a, the carbonyl contents of the control and E64 groups demonstrated a significant increase from 0 to 0.5 mM H_2_O_2_ (*p* < 0.05) followed by a sudden significant decrease at 1 mM H_2_O_2_ and then kept at the similar low level with no significant difference under 1–100 mM H_2_O_2_ (*p* > 0.05). Interestingly, the higher carbonyl content of myofibrillar protein at 0.5 mM H_2_O_2_ than that at 1–10 mM H_2_O_2_ was corresponded to the lower MHC intensity at 0.5 mM H_2_O_2_ than that at 1–10 mM H_2_O_2_. The increase of carbonyl contents was because the amino acid residuals could form carbonyl derivatives due to hydroxyl-radical-induced oxidation conversion, which was similarly reported by Lu, Zhang, Li, and Luo [12] in the myofibrillar protein of bighead carps exposed to a hydroxyl radical-generating system. The carbonyl contents also probably decreased with continued oxidation because of its attendance with electron-dense groups such as amines, creating crosslinked protein aggregates in advanced reactions afterwards [38]. In addition, the samples in the control group demonstrated significantly higher carbonyl contents than that in E64 group at 0.1 and 50 mM H_2_O_2_ (*p* < 0.05), which was probably because the myofibrillar protein in the control group within cathepsins could result in more protein degradation and thus the generated protein fragmentation formed more carbonyl derivatives under hydroxyl radical systems.

#### 3.3.2. Total Sulfhydryl Content

As shown in Figure 3b, there was no significant difference between the total sulfhydryl contents of the control group treated with 0–5 mM H_2_O_2_, which was followed by a considerable decrease from 10–100 mM H_2_O_2_ (*p* < 0.05), while the total sulfhydryl contents of E64 group fluctuated between 0.16 to 0.18 mmol/g protein at 0–50 mM H_2_O_2_ and declined significantly at 100 mM H_2_O_2_ (*p* < 0.05). The significant decrease of the total sulfhydryl content was attributed to oxidation caused by increasing hydroxyl radicals, which was also observed in porcine myofibrillar protein gels under hydroxyl radicals [39]. In addition, the control group demonstrated significantly higher total sulfhydryl contents than the E64 group at 0 mM H_2_O_2_, which was probably because cathepsins existing in the control group resulted in more protein degradation and thus generated more sulfhydryl derivatives, or the cathepsins existing in the control group contained sulfhydryl derivatives as active sites. The result indicated a greater decrease of total sulfhydryl content observed in the control group than that in the E64 group at 10–50 mM H_2_O_2_ compared with 0 mM H_2_O_2_, which suggested that myofibrillar protein with cathepsins were more easily affected by hydroxyl-radical-induced oxidation, which was consistent with the results of the carbonyl contents.

### 3.4. Chemical Interactions of Myofibrillar Protein (Before Heating) and Gel (After Heating)

**Figure 4:**
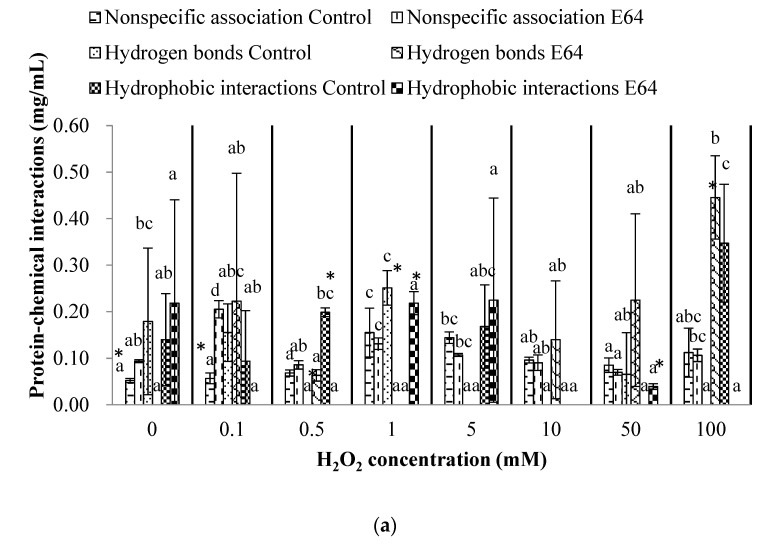

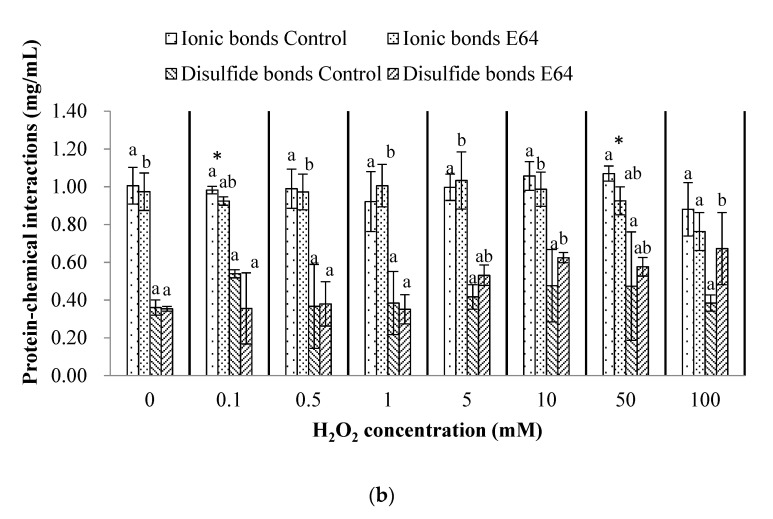
Nonspecific association, hydrogen bonds, and hydrophobic interaction (**a**); ionic bonds and disulfide bonds (**b**) of myofibrillar protein from bighead carps under different H_2_O_2_ concentrations. The same lowercase letter indicates no significant differences between samples of the control/E64 group under different H_2_O_2_ concentrations. The asterisk indicates significant difference between samples at control and E64 groups under the same H_2_O_2_ concentration (*p* < 0.05).

The chemical interactions, including nonspecific association, ionic bonds, hydrogen bonds, hydrophobic interaction, and disulfide bonds of myofibrillar protein, were established in Figure 4. For myofibrillar protein, the nonspecific association, hydrogen bonds and hydrophobic interaction were all less than 0.3 mg/mL, except for the hydrogen bonds of myofibrillar protein in the E64 group (0.45 mg/mL) and the hydrophobic interaction of myofibrillar protein in the control group (0.35 mg/mL) at 100 mM H_2_O_2_. Thus, we mainly focused on the changes of ionic bonds (more than 0.5 mg/mL) and disulfides (more than 0.3 mg/mL) of myofibrillar protein. Zhang et al. [40] also found the dominated proportion of ionic bonds in surimi paste compared with hydrogen bonds and hydrophobic interaction. The ionic bonds of the E64 group were fluctuant during 0–50 mM H_2_O_2_ and decreased significantly to 0.76 mg/mL at 100 mM H_2_O_2_ (*p* < 0.05). In addition, the disulfide bonds of the E64 group increased significantly to 0.67 mg/mL at 100 mM H_2_O_2_ (*p* < 0.05), which was probably caused by the hydroxyl radicals oxidation, corresponding to the decrease of total sulfhydryl contents with the increasing H_2_O_2_ concentration. The increase of disulfide bonds and decrease of ionic bonds of E64 myofibrillar protein implied that the extensive oxidation (100 mM H_2_O_2_) inhibited the formation of ionic bonds and promoted the formation of disulfide crosslinking between myofibrillar proteins. For the control group, no significant changes in ionic bonds or disulfide bonds were observed under different H_2_O_2_ concentrations. In addition, the ionic bonds of the control group was greatly higher than that of E64 at 0.1 and 50 mM H_2_O_2_, which was probably attributed to more charged peptides residues from the myofibrillar protein degradation and fragmentation caused by cathepsin hydrolysis in the control group at 0.1 and 50 mM H_2_O_2_.

**Figure 5:**
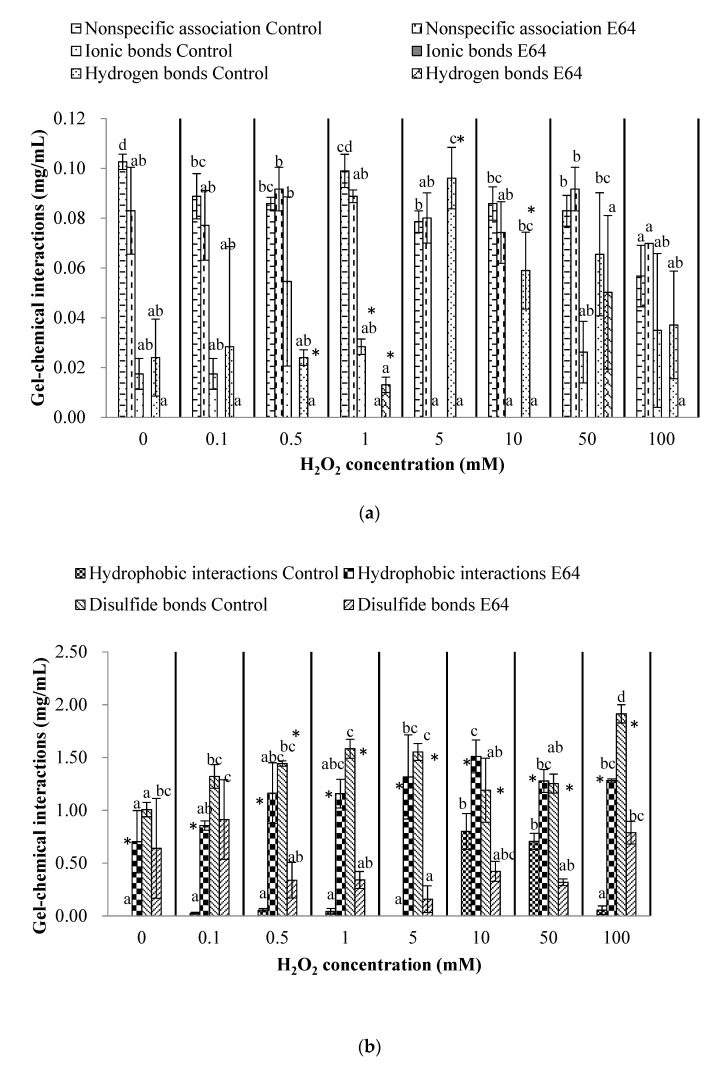
Nonspecific association, ionic bonds, and hydrogen bonds (**a**); hydrophobic interaction and disulfide bonds (**b**) of gels from bighead carps under different H_2_O_2_ concentrations. The same lowercase letter indicates no significant differences between samples of control/E64 group under different H_2_O_2_ concentrations (the ionic bonds of E64 were 0 mg/mL so the data and lowercase letter were not shown in (**a**). The asterisk indicates significant difference between samples at control and E64 groups under the same H_2_O_2_ concentration (*p* < 0.05).

The ionic bonds, hydrogen bonds, hydrophobic interaction, and disulfide bonds of myofibrillar protein demonstrated dramatic changes to form gels after thermal treatments in Figure 5. The ionic bonds and hydrogen bonds between gel protein molecules almost disappeared after heating. Heat improved the unfolding of the protein structure and resulted in the exposure of hydrophobic groups [41]. Liu et al. [42] found that thermal treatment contributed to the formation of disulfide bonds in heat-induced fish and pork protein gel. Therefore, the hydrophobic interactions and disulfide bonds were the dominant chemical interaction for gels. The hydrophobic interactions of gels in the control group increased significantly from the initial 0 to 0.80 and 0.71 mg/mL at 10 and 50 mM H_2_O_2_, respectively, and then decreased to 0.05 mg/mL at 100 mM H_2_O_2_ (*p* < 0.05). In addition, the gels of the E64 group demonstrated significantly increased hydrophobic interactions during 0 to 100 mM H_2_O_2_, ranging from 0.70 to 1.51 mg/mL, which were significantly higher than that of control group (*p* < 0.05). Correspondingly, the disulfide bonds of the E64 group gels were significantly lower than that of the control group (*p* < 0.05). The disulfide bonds of E64 gels fluctuated during oxidation, ranging from 0.16 to 0.91 mg/ mL, while the disulfide bonds of control gels increased, firstly, from 0 to 5 mM H_2_O_2_ and then decreased to 1.19 and 1.25 mg/mL at 10 and 50 mM H_2_O_2_, respectively, followed by an increase to 1.91 mg/mL at 100 mM H_2_O_2_ (*p* < 0.05). These results indicated that myofibrillar protein in the control group within cathepsin hydrolysis preferred to form gels with lower hydrophobic interactions and higher disulfide bonds after heating, which was probably because the cathepsin hydrolysis in the control group could lead to more protein degradation. Thus, more protein fragmentation was susceptible to hydroxyl radicals and then formed more disulfides; then, the hydrophobic interactions, another dominant chemical force, were less.

### 3.5. Gel Properties

#### 3.5.1. Gel Strength

**Figure 6:**
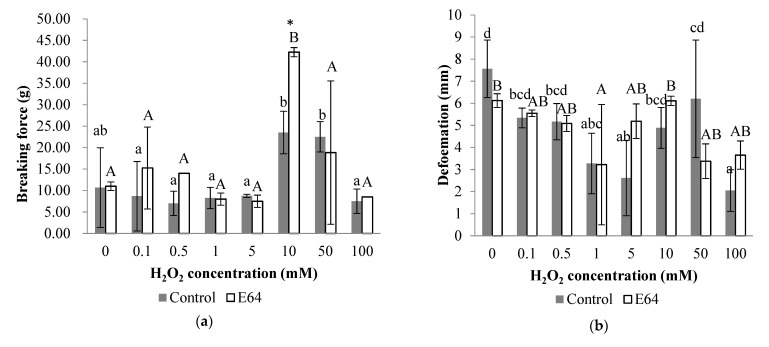

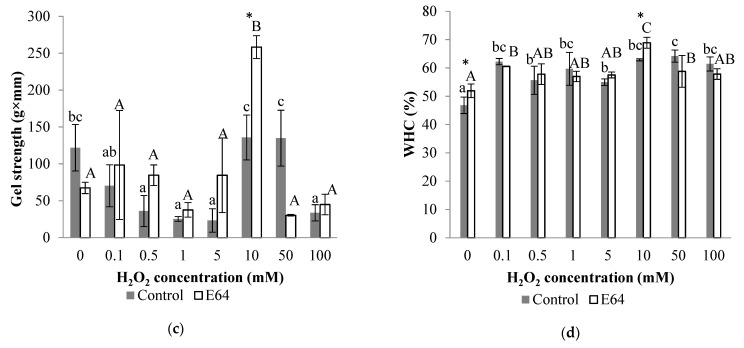
Gel properties of myofibrillar protein gels from bighead carps under different H_2_O_2_ concentrations: (**a**) breaking force; (**b**) deformation distance; (**c**) gel strength; (**d**) WHC. The same lowercase letter indicates no significant differences between samples of control group under different H_2_O_2_ concentrations. The same capital letter indicates no significant differences between samples of E64 group under different H_2_O_2_ concentrations. The asterisk indicates significant difference between samples at control and E64 groups under the same H_2_O_2_ concentration (*p* < 0.05).

The breaking force of the control and E64 groups increased with H_2_O_2_ concentration and reached the peak level 23.5 and 42.3 g at 10 mM H_2_O_2_, respectively, followed by a significant decline at 100 mM H_2_O_2_, as shown in Figure 6a (*p* < 0.05). In addition, the breaking force of the control group was significantly lower than that of the E64 group at 10 mM H_2_O_2_ (*p* < 0.05), which could be attributed to the effects of the cathepsins existing in the control group on myofibrillar protein degradation and thus resulting in a loss of breaking force.

The deformation distance of the control group demonstrated a decrease, firstly, and reached the lowest value at 5 mM H_2_O_2_, then increased at 10–50 mM H_2_O_2_ followed by a significant decline at 100 mM H_2_O_2_ in Figure 6b (*p* < 0.05). The deformation distance of the E64 group demonstrated a similar changing trend with the control group, which demonstrated the lowest value at 1 mM H_2_O_2_ and then an increase at 10 mM H_2_O_2_ in Figure 6b. In addition, there was no significant difference between deformation distance of the control and E64 groups during oxidation (*p >* 0.05).

Based on the results of breaking force and deformation distance, the gel strength of the E64 group was higher at 10 mM H_2_O_2_ than other H_2_O_2_ concentrations (*p* < 0.05) in Figure 6c. While the gel strength of control group decreased from 0 to 5 mM H_2_O_2_, and then increased to 10 and 50 mM H_2_O_2_ and decreased again at 100 mM H_2_O_2_ (*p* < 0.05). The higher gel strength of the control and E64 groups at 10 mM H_2_O_2_ was similar to the myofibrillar protein gels from bighead carp under hydroxyl radicals, which demonstrated higher gel texture under mild oxidation than other oxidation conditions [12]. In addition, the significant higher gel strength in the E64 group (258.26 g·mm) than that in the control group (135.71 g·mm) occurred at 10 mM H_2_O_2_, which was corresponding to the result of breaking force, also revealed that the cathepsins existing in the control group had destructive effects on gel strength due to their hydrolysis on myofibrillar protein. Liu, Yin, Zhang, Li, and Ma [6] also reported that the gel strength, breaking force, and deformation were significantly decreased when treated with cathepsin B and L. Cao et al. [43] also found that the addition of cathepsin L reduced the gel strength of the surimi gel compared to the control group without cathepsin L.

#### 3.5.2. WHC

WHC is a quantitative indication of the amount of water retained within the structure of the protein gel network, which could reflect the spatial structure of protein gels [12]. As shown in Figure 6d, the E64 and control groups all demonstrated higher WHC under oxidation than that at 0 mM H_2_O_2_, and the control and E64 groups, especially, reached their highest WHC at 50 mM and 10 mM H_2_O_2_, which were corresponded to the higher gel strength of the control and E64 gels at 50 mM and 10 mM H_2_O_2_, respectively. The promotion effect of oxidation on WHC was also found in myofibrillar protein gels under 0.1–5 mM H_2_O_2_ in other reports [12]. In addition, a significantly higher WHC of the E64 group at 0 and 10 mM H_2_O_2_ was observed than that of the control group. This result could be ascribed to the destructive effects of cathepsin hydrolysis on myofibrillar proteins, particularly on MHC, as shown in SDS-PAGE, which led to a weakened surimi gel with poor WHC, as described by Tang et al. [44].

## 4. Discussion

The effects of oxidation caused by increasing hydroxyl radicals on myofibrillar protein were expressed by the results in the E64 group, which demonstrated increased and then decreased carbonyl contents, declined total sulfhydryl contents of myofibrillar protein, decreased intensity bands of MHC and actin, decreased ionic bonds, and increased disulfide bonds between the molecular myofibrillar protein. Chen et al. [45] suggested that hydrophobic interactions and disulfide bonds are the main chemical interactions for stabilizing the gel structure. In present study, the breaking force and gel strength of E64 gels reached the highest level at 10 mM H_2_O_2_ probably because the E64 gels had higher hydrophobic interactions (1.51 mg/mL) and moderate disulfide bonds (0.42 mg/mL) between gel molecules at 10 mM H_2_O_2_. Besides, the oxidation caused by 10 mM H_2_O_2_ resulted in higher WHC of E64 gels, also probably due to the higher hydrophobic interaction of gels at 10 mM than that at 0 mM H_2_O_2_, which contributed to the formation of the stable three-dimensional structure of gel [46], and thus facilitated the gel WHC. In addition, moderate disulfide crosslinking of myofibrillar protein at 10 mM H_2_O_2_ would reduce the empty spaces and change the aggregate gel structure into a fine gel network to some degree [47], consequently having a good WHC.

The cathepsin L played a more important role in hydrolysis due to its higher activity than cathepsin B in Figure 1. The oxidation caused by hydroxyl radicals reduced cathepsin B and cathepsin L activities, which would weaken the proteolysis and sensitivities to myofibrillar protein with an increasing H_2_O_2_ concentration. The effects of cathepsin hydrolysis on myofibrillar proteins were evaluated through the difference between the results of the control and E64 groups. Significantly higher carbonyl contents of myofibrillar protein in the control group than that in the E64 group were observed at 0.1 and 50 mM H_2_O_2_. By coincidence, higher ionic bonds between molecular myofibrillar proteins in the control group than that in the E64 group also occurred at 0.1 and 50 mM H_2_O_2_. In addition, the control group demonstrated significantly higher total sulfhydryl contents than the E64 group at 0 mM H_2_O_2_. Lower intensity of MHC and actin bands in the control SDS-PAGE than that in the E64 group during 0–50 mM H_2_O_2_ also confirmed the cathepsin hydrolysis, as the susceptibility of MHC and actin to proteolysis by cathepsins B and L in vitro studies [35,36,37]. These results revealed that more myofibrillar protein degradation and fragmentation occurred in the control group during 0–50 mM H_2_O_2_ due to cathepsin proteolysis, which probably contributed to the deterioration of gel strength and WHC. Consequently, the E64 group demonstrated notably higher gel strength and WHC at 10 mM H_2_O_2_ than the control group. Liu, Yin, Zhang, Li, and Ma [6] and Hu et al. [8] also reported the destructive effects of cathepsins on gel properties involved with surimi modori. One reason for higher gel strength and WHC in the E64 group than the control group could probably be attributed to less myofibrillar protein degradation in the E64 group than that in the control group, as shown in SDS-PAGE profile. The MHC, as the most critical protein for the formation of gel network structure, is sensitive to cathepsin hydrolysis. The greater degradation of MHC caused by cathepsins in the control group therefore resulted in lower gel strength and WHC. Another reason for higher gel strength and WHC in the E64 group than the control group was probably because of higher hydrophobic interaction between the gel protein molecules of the E64 group than the control group (*p* < 0.05). Liu et al. [48] proved that the main chemical interactions in stabilizing the surimi gel network were hydrophobic interactions. Higher hydrophobicity could literally strengthen the binding of the proteins and the formation of stable three-dimensional structure of gel, which can promote gel strength and WHC. The cathepsin hydrolysis resulted in the degradation and fragmentation of myofibrillar protein, therefore making an increase of solubility and hydrophilicity of proteins; thus, the hydrophobicity was lower, as shown in the control gels than the E64 gels. The third reason for higher gel strength and WHC in the E64 group than the control group was due to the E64 gels having a moderate level of disulfide bonds (0.42mg/mL) between gel protein molecules, instead of a higher level of disulfide bonds (1.19mg/mL) in the control group (*p* < 0.05). A moderate content of disulfide crosslinks would promote gel strength, while high disulfide crosslinks were harmful to gel texture [12]. When the gel strength is high, the trapped moisture is not easily extruded, which implies higher WHC [49]. In addition, there was a more decline in the total sulfhydryl content observed in the control group than that in the E64 group at 10 mM H_2_O_2_, which could be caused by cathepsin hydrolysis as described above, and was consistent with higher disulfide bonds of the control gels than that ofthe E64 gels at 10 mM H_2_O_2_.

## 5. Conclusions

Mild oxidation caused by hydroxyl radicals (10 mM H_2_O_2_) made a better gel strength and WHC from bighead carp myofibrillar protein. In addition, cathepsin B and L activities decreased sharply with an increasing H_2_O_2_ concentration but their effects on myofibrillar protein degradation still existed under 0.1–50 mM H_2_O_2_. Besides the protein degradation, the cathepsins also resulted in lower hydrophobic interaction and moderate disulfides bonds between gel protein molecules, thus leading to lower gel strength and WHC of myofibrillar protein gels under mild oxidation. Therefore, we should preclude the cathepsins as much as possible even when the cathepsins just have a low level of activity in surimi to avoid the destructive effects on gel qualities.

## Data Availability

Data available on request.
